# Does Anzer Propolis Have a Protective Effect on Rabbit Spinal Cord Ischemia/Reperfusion Injury?*

**DOI:** 10.21470/1678-9741-2020-0430

**Published:** 2022

**Authors:** Murat Günday, Zülfükar Kadir Saritaş, Hasan Hüseyin Demirel, Aziz Bülbül, Tuba Berra Saritaş, Fatma Görücü, Necip Becit

**Affiliations:** 1 Department of Cardiovascular Surgery, Afyonkarahisar Health Sciences University, Faculty of Medicine, Afyonkarahisar, Turkey.; 2 Department of Surgery, Afyon Kocatepe University, Faculty of Veterinary Medicine, Afyonkarahisar, Turkey.; 3 Bayat Laborant and Veterinary Health Division, Afyon Kocatepe University, Afyonkarahisar, Turkey.; 4 Department Of Physiology, Muğla Sıtkı Koçman University, Milas Veterinary Faculty, Muğla, Turkey.; 5 Department of Anesthesiology and Reanimation, Afyonkarahisar Health Sciences University, Faculty of Medicine, Afyonkarahisar, Turkey.

**Keywords:** Propolis, Ischemia, Reperfusion, Rabbits, Animals, Biomarkers, Peroxidase, Solubility

## Abstract

**Introduction:**

In this study, Anzer propolis, which can only be obtained from the Eastern Black Sea region in Turkey, is studied for its effect on spinal cord ischemia/reperfusion injury.

**Methods:**

A total of 12 healthy male New Zealand White rabbits with an average weight of 3.0 to 3.5 kg were separated into two blind and randomized groups: the ischemia/reperfusion group (n=6) and the treatment group (n=6). Each rabbit in the treatment group was given a dose of 100 mg/kg of ethanol-dissolved Anzer propolis orally 1 hour before surgery. Blood samples were examined at the 0^th^ hour and postoperatively at the 24^th^ and 48^th^ hours. Tissue samples were taken at the 48^th^ hour during the sacrification.

**Results:**

There was a statistically significant difference between the two groups in terms of postoperative Tarlov scoring (*P*=0.012). There was a difference between the two groups in terms of the blood levels of interleukin-6 (IL-6) and tumor necrosis factor-alpha (TNF-α) at the 48^th^ hour, myeloperoxidase (MPO) at the 24^th^ and 48^th^ hours, ischemia-modified albumin (IMA) at the 24^th^ hour, and intercellular adhesion molecule-1 (ICAM-1) and total oxidant status (TOS) at the 48^th^ hour (*P*<0.005). There was also a difference between the two groups in terms of apoptotic index data obtained with the terminal deoxynucleotidyl transferase (TdT)‐mediated dUTP nick‐end labelling (TUNEL) method in the histopathological examination (*P*=0.001). In the transmission electron microscopic (TEM) analysis, while ischemia/reperfusion group generally had axon-myelin separation, axoplasmic dissolution and myelin separation, the propolis treatment group had normal myelin sequencing.

**Discussion:**

In our study, after biochemical, histopathological, ultrastructural and neurological functional examination, it was demonstrated that Anzer propolis has sufficient neuroprotective effect on spinal cord ischemia/reperfusion injury in rabbits.

**Table:** 

Abbreviations, acronyms & symbols				
**ACTH** **ANOVA** **CAPE** **DAB** **ELISA** **HE** **ICAM-1** **IL-6**	**= Adrenocorticotropic hormone** **= Analysis of variance** **= Caffeic acid phenethyl ester** **= Diaminobenzidine** **= Enzyme-linked immunosorbent assay** **= Hematoxylin-eosin** **= Intercellular adhesion molecule-1** **= Interleukin-6**		**IMA** **MPO** **ROS** **TAS** **TEM** **TNF-α** **TOS**	**= Ischemia-modified albumin** **= Myeloperoxidase** **= Reactive oxygen species** **= Total antioxidant status** **= Transmission electron microscopic** **= Tumor necrosis factor-alpha** **= Total oxidant statu**s

## INTRODUCTION

Spinal cord injury continues to be an important problem after descending and thoracoabdominal aortic surgery. The most serious complication after spinal cord injury may be paraplegia. The rate of paraplegia after thoracoabdominal aortic surgery has been reported as 2.4-40% ^[1]^.

Spinal cord injury occurs due to ischemia/reperfusion damage created by cross-clamp during the surgical procedure. Two main pathophysiological mechanisms are responsible for this event ^[2]^. In the first stage, the hypoxia that occurs in neurons after ischemia is called a primary injury. Spinal cord neurons are sensitive to ischemic injuries because of their high energy demand. Primary morphological changes include disruption of tissue integrity, damage to blood vessels and axons, edema, and disruption of cells. Following the primary damage, secondary damage, which includes some pathophysiological changes, such as ischemia, ion infiltration, production of oxygen radicals, and lipid peroxidation, occurs in hours and days ^[3-5]^. However, reperfusion that occurs especially after prolonged ischemia paradoxically causes tissue damage. Reactive oxygen species (ROS) play a key role in the secondary injury caused by reperfusion ^[6]^. For this reason, various drugs or chemicals have been used to prevent ROS formation in ischemia/reperfusion injury in different tissues, including the spinal cord ^[7]^.

Propolis is a bee product that has become popular for the last few years. It has a high antioxidant composition ^[8]^. It has antineurotoxic, antiviral, antibacterial, anticancer and antioxidant effects that have been reported in the literature for cardiovascular diseases, diabetes, and cancer treatments ^[9-11]^.

The chemical composition of propolis varies depending on the vegetation, climate, season and environmental conditions of the area it is collected.

The aim of our study is to investigate the effects of Anzer propolis, which can only be obtained from the Eastern Black Sea region in Turkey, on histopathological changes in the spinal cord after ischemia/reperfusion injury, electron microscopic analysis, antioxidant status, lipid peroxidation and clinical improvement.

## METHODS

All animals were given humane care in accordance with the criteria of the *Guide for the Care and Use of Laboratory Animals*, published by the US National Institutes of Health. The study started with the approval given by the Local Ethics Committee for Animal Experiments at Afyon Kocatepe University (approved on 20/08/2019 under number AKÜ HADYEK-77-19).

The Anzer propolis that we used in our study was analyzed by spectrometry method in the laboratories of the Hacettepe University Advanced Technology and Research and Application Centre.

### Experiment Design

#### Experimental Group

The rabbits were housed in individual polypropylene cages at least 7 days before the beginning of the experiment and given *ad libitum* access to food and water with a natural day-night cycle. All animal manipulations were performed according to the *Guide for the Care and Use of Laboratory Animals*, published by the US National Institutes of Health (NIH publication No. 85-23, revised in 1996).

A total of 12 healthy male New Zealand White rabbits, with an average weight of 3.0 to 3.5 kg, were separated into two blind and randomized groups: ischemia/reperfusion group (I/R, n=6, group 1) and treatment group (Pr, n=6, group 2). Each rabbit in the treatment group was given orally 100 mg/kg of Anzer propolis dissolved in ethanol 1 hour before surgery ^[12]^. Ischemia/reperfusion and treatment groups were subjected to 30 minutes of cross-clamp and 1 hour of reperfusion. Blood samples of the subjects were collected just before the surgical procedure (hour 0), and at 24 and 48 hours after surgery. Tissue samples were collected during the sacrification process at 48 hours.

#### Anesthesia and Monitoring

In all subjects, anesthesia was administrated with ketamine hydrochloride (Ketas, Parke-Davis, Eczacıbaşı, Istanbul, Turkey) 50 mg/kg and xylazine hydrochloride (Rompun, Bayer, Istanbul, Turkey) 5 mg/kg, intramuscularly. Subjects were kept at a pH between 7.35 and 7.45 (ABL 5, radiometer, Kopenhagen). Oxygen support was provided to all rabbits, and arterial blood pressure was continuously monitored after catheterization of femoral artery under general anesthesia by bedside monitor (multichannel bedside monitor Petaş KMA 800, Petaş A.Ş. Ankara, Turkey), and mean arterial blood pressure was kept around 80 mmHg. Body temperatures were monitored by rectal probs connected to the same monitor and were maintained at around 38.5-39.5 °C using heated blankets. Hypothermia was never applied to any of the subjects ^[13]^.

#### Surgical Technique

The subjects were operated in a supine position under sterile conditions following anesthesia. After sterile surgical condition, a median laparotomy was performed. The intestines were deviated to the right and the retroperitoneal region was reached. The same surgeon reached the retroperitoneal region by microsurgery and dissected the abdominal aorta about 1 cm inferior to the left renal artery and about 1 cm superior to the bifurcation from the surrounding tissues, and sutured it around. Anticoagulation was achieved with 150 U.kg^-^¹ heparin applied intravenously, and the aortic clamp was applied to the subjects. It was examined manually that the pulse in the femoral artery had completely disappeared. The clamp was applied for 30 minutes and removed at the end of the period. I/R group and Pr Group were subjected to reperfusion for 1 hour ^[13]^. The laparotomy incision was closed in two layers as abdominal muscles and the skin, with 3/0 Prolene sutures following bleeding control.

Subjects were awakened and taken into their cages. They were kept under observation in terms of neurological and vital functions. After 48 hours, individuals in I/R and Pr groups were sacrificed with IV application of a high dose of thiopental sodium (Pental Sodium, 0.5 g, I.E. Ulagay, Turkey).

#### Biochemical Analysis

Venous blood was collected 3 times from all groups, just before the surgical procedure (0^th^ hour) and at the 24^th^ and 48^th^ hours postoperatively. Blood gas and complete blood count analyses were performed. The serum samples were stored at -80 °C. Serum samples of IL-1 (rabbit interleukin-1, ELISA kit, catalog no: YLA0081RB, 96 tests) and IL-6 (rabbit interleukin-6, ELISA kit, catalog no: YLA0011RB, 96 tests in serum samples), TNF-α (rabbit tumor necrosis factor-alpha, ELISA kit, catalog no: YLC0025RB, 96 tests), total antioxidant status (TAS) (Cayman 709001, antioxidant assay kit), total oxidant status (TOS) (Rel Assay Diagnostics), intercellular adhesion molecule-1 (ICAM-1) (rabbit intracellular adhesion molecule-1, ELISA kit, catalog no: YLA0064RB, 96 tests), myeloperoxidase (MPO) (rabbit myeloperoxidase, ELISA kit, catalog no: YLA0057RB, 96 tests), IMA (rabbit ischemia modified albumin, ELISA kit, catalog no: YLA00164RB, 96 tests) were measured in an ELISA device (MVGt Lambda Scan 200, Bio-Tek Instrument, VT) with rabbit ELISA kits.

#### Postoperative Neurological Evaluation

The motor functions of the hind limbs were evaluated according to Tarlov criteria at 6, 24, and 48 hours after reperfusion, by a researcher who did not know the treatment applied beforehand ^[14]^.

#### Tarlov Score

According to Tarlov score, 0 was used if there was no movement in the lower limbs (complete paraplegia), 1 for slight movements, 2 if the animal could sit with help, 3 if the animal could sit alone, 4 if the animal could walk but abnormally, 5 if the animal could walk normally.

#### Histopatologic Examination

Subjects were euthanized with high-dose thiopental. By performing laminectomy at L2-5 levels, samples were taken from the spinal cord without damaging it and examined by using 10% formaldehyde and glutaraldehyde solutions. In histopathological examination, apoptosis and tissue necrosis were evaluated.

#### Hematoxylin-Eosin Staining Protocol

Spinal cord tissue samples were examined in 10% buffered formalin solution for histopathological examinations using the hematoxylin-eosin (HE) staining method ^[15]^. The stained preparations were examined under a binocular light microscope (Nikon, Eclipse Ci, Tokyo, Japan). Microscopic images were taken from the required preparations (Nikon DS F1, microscopic digital camera systems, Tokyo, Japan).

#### TUNEL Protocol

ApopTag^®^ Peroxidase In Situ Apoptosis Detection kit (Milipore S7101, USA) was used for this technique. The sections were kept in a 60 °C oven for staining for one night, then the transparency process was performed with three exchanges of xylene every 30 minutes. Then, rehydration was achieved by decreasing alcohol series and kept in distilled water for 10 minutes. The area to be stained was drawn with PapPen. Sections were incubated for 15 minutes with proteinase K. Then, the sections were washed 5 minutes 3 times with buffer solution. After each wash, water overflow around the tissue was removed with blotting paper. The sections were then incubated with 3% hydrogen peroxide for 10 minutes to block endogenous peroxidase activity. The sections were washed again with buffer solution and then incubated with equilibration buffer for 10 minutes. The sections were then kept in the oven for 1 hour at 37 ºC with Tdt enzyme (770 µL of reaction buffer + 330 µL of Tdt enzyme). After 1 hour, the sections were incubated with working strength stop/wash buffer (100 µL of stop wash buffer + 3,400 µL of distilled water) for 10 minutes. The sections washed with buffer solution were stained with diaminobenzidine (DAB) to determine the visibility of the terminal deoxynucleotidyl transferase (TdT)-mediated dUTP nick-end labelling (TUNEL) reaction. For this, 30 µL of DAB substrate and 1,470 µL of DAB dilution buffer were mixed and left to rest for half an hour before being used in a dark place. The DAB solution prepared in the sections was placed and kept for approximately 4 minutes. The preparations were washed with distilled water. The slide staining base was prepared by dripping methyl green on the slides and waiting 10-15 seconds. After removing the excess stain, the lamellas were passed through alcohol and xylene series without being dried, then closed with Entellan^®^ (UN 1866, Merck, Darmstadt, Germany).

#### Transmission Electron Microscopic Analysis

The samples collected during surgery were taken for primary fixation at 4 °C for 24 hours in 2.5% glutaraldehyde containing 0.1 M phosphate buffer, and then washed 3 times for 15 minutes with phosphate buffer. The blocks obtained were cut at 700 nm thickness with an ultramicrotome (Leica Ultracut R) and stained with toluidine blue. After determining the areas to be displayed in transmission electron microscopic (TEM), every subject was scored according to the criteria shown in Table 1 ^[16]^.

**Table 1:** Electron microscopic scoring criteria for specimens.

		I/R group	Pr group	*P*
Intracellular edema		4.25±0.96	2.00±0.00	0.011
None	0			
+	1			
++	2			
**Axonal degeneration**				
Normal myelin layers	0			
Splitting of myelin layers	1			
Fragmentations of myelin layers	2			
Honeycomb appearance	3			
**Axonal/myelin degeneration**				
None	0			
Yes	1			
**Mitochondrial injury**				
Normal	0			
Mild	1			
Severe	2			

I/R=ischemia/reperfusion; Pr=propolis

### Statistical Analysis

The results obtained in the research were carried out with the SPSS 16.0 software (SPSS Inc., Chicago, IL, USA). The Kolmogorov-Smirnov test was always used to determine whether the data distribution was normal. The one-way ANOVA test was used for blood count and biochemical analysis, which are among the results obtained from the research. Mann-Whitney U test and independent t-test were used in the analysis of other continuous variables. The results were expressed as mean±standard deviation. *P*<0.05 was accepted for statistical significance.

## RESULTS

### Propolis Analysis

It has been identified that the Anzer propolis used in the research had the highest levels of ethyl oleate (10,23%) and 4H-1-benzopyran-4-one, 5-hydroxy (9,07%) (Table 2).

**Table 2:** Spectrometry analysis of propolis.

Compounds	% ratio
2-methoxy-4-vinylphenol	3.30
6-phenyl-5-hexenoic acid, methyl	1.02
Guaiol	0.40
Dodecanoic acid ethyl ester	1.11
Agarospirol	0.95
Bicyclo[4.4.0]dec-1-ene, 2-isopr	0.88
2-naphthalenemethanol, decahydro	4.60
2-naphthalenemethanol, 1,2,3,4,4	4.31
Naphthalene, 1,2,4a,5,6,8a-hexah	0.74
(1R,7S,E)-7-isopropyl-4,10-dimet.	0.39
Alpha-bisabolol	3.79
Benzyl benzoate	1.83
7-(2-hydroxypropan-2-yl)-1,4a-di	1.01
(-)-spathulenol	0.38
(1R,4aR,7R,8aR)-7-(2-Hydroxyprop	4.72
Tetradecanoic acid, ethyl ester	0.63
Hexadecanoic acid, ethyl ester	4.26
4b,8-dimethyl-2-isopropylphenant	0.10
(E)-cinnamyl benzoate	1.53
Benzyl cinnamate	2.98
Heptadecanoic acid, ethyl ester	0.56
1-propene-1,2,3-tricarboxylic ac	0.28
Linoleic acid ethyl ester	2.41
Ethyl oleate	10.23
2-propenoic acid, 3-(4-hydroxy-3..	1.72
Benzaldehyde, 4,6-dimethoxy-2,3-.	0.77
Octadecanoic acid, ethyl ester	1.74
Trans-ferulic acid	1.54
Butyl citrate	2.44
(1aR,4aS,8aS)-4a,8,8-trimethyl-1..	2.44
2-phenanthrenol, 4b,5,6,7,8,8a,9.	1.72
Ferruginol	0.55
1-phenanthrenemethanol, 1,2,3,4,...	0.64
4H-1-benzopyran-4-one,2,3-dihydro	6.35
Cinnamyl cinnamate	3.58
Eicosanoic acid, ethyl ester	2.97
Cedrol	1.45
2-(acetoxymethyl)-3-(methoxycarb..	0.55
Pyrido[2,3-d]pyrimidine, 4-phenyl-	0.45
Benzyl (E)-ferulate	4.44
4H-1-benzopyran-4-one, 5-hydroxy..	9.07
2-bromo-4,5-dimethoxycinnamic acid	0.53
(1R,4aR,4bS,7R,10aR)-1,4a,7-Trim...	3.02

### Biochemical Results

There was a difference between the two groups in terms of 48^th^ hour IL-6, 48^th^ hour TNF-α, 24^th^ hour MPO, 48^th^ hour MPO, 24^th^ hour IMA, 48^th^ hour ICAM-1, and 48^th^ hour TOS values (*P*=0.022, 0.040, 0.037, 0.023, 0.015, 0.012, and 0.002, respectively). There was no difference in terms of other parameters (Table 3).

**Table 3:** Biochemical analysis results in groups (mean±SD).

Parameter/Group	0^th^ hour	24^th^ hour	48^th^ hour
**IL-1 I/R**	4,8900±,23956c	10,3350±,53293a	7,4367±,52164b
**Pr**	4,9683±,28891b	9,3500±,50484a	8,3883±,38178a
***P***	0.763	0.303	0.298
**IL-6 I/R**	43,5933±1,81007c	149,6283±15,75694a	81,0450±4,68628b
**Pr**	39,0800±2,90564c	111,2600±3,28355a	59,6033±5,26723b
***P***	0.168	0.066	0.022
**TNF-α I/R**	21,4983±,84374	255,711111183±17,47462	145,1700±17,44557
**Pr**	24,4500±3,57613	246,3683±32,18286	85,2800±10,33481
***P***	0.425	0.759	0.040
**MPO I/R**	4,2133±,38473b	5,9467±,55778ab	7,5783±,50105a
**Pr**	4,5233±,24887b	4,6183±,18816b	5,7117±,27411a
***P***	0.548	0.037	0.023
**IMA I/R**	1,3820±,06135b	1,6760±,07814a	1,8500±,08438a
**Pr**	1,4380±,05970b	1,5340±,04632b	1,7700±,04494a
***P***	0.548	0.015	0.642
**ICAM-1 I/R**	187,5667±6,12962c	357,3117±24,00794a	252,1100±15,60926b
**Pr**	197,2983±8,85513b	320,6283±38,75100a	205,7600±14,84168b
***P***	0.339	0.219	0.012
**TOS I/R**	4,8483±,22494b	6,0633±,26670a	6,6767±,42214a
**Pr**	5,0733±,25741	5,5700±,19471	4,8633±,18903
***P***	0.599	0.099	0.002
**TAS I/R**	6,9383±,41051	7,1500±,54968	7,3517±,35899
**Pr**	7,3467±,51261	7,2533±,23937	7,7167±,21066
***P***	0.061	0.833	0.241

ICAM-1=internal adhesion molecule-1; IL-1=interleukin 1; IL-6=interleukin 6; IMA=ischemia-modified albumin; I/R=ischemia/reperfusion; MPO=myeloperoxidase; Pr=propolis; TAS=total antioxidant capacity; TNF-α=tumor necrosis factor-alpha; TOS=total oxidant capacity

In terms of blood count data between the two groups, there was a difference between postoperative 24^th^ hour lymphocyte, granulocyte and 48^th^ hour monocyte (*P*=0.038, 0.011, and 0.008, respectively). There was no difference between the other parameters.

### Neurological Results

In the I/R group, 5 rabbits received 1 point, and 1 rabbit received 2 points after the procedure. In the Pr group, it was determined that 3 animals were walking (all 5 points). Two rabbits received 3 points and 1 rabbit received 1 point. A statistically significant difference was found between the two groups in terms of postoperative Tarlov scoring (*P*=0.012).

### Results of Histopathological Analysis

**Fig. 1:**
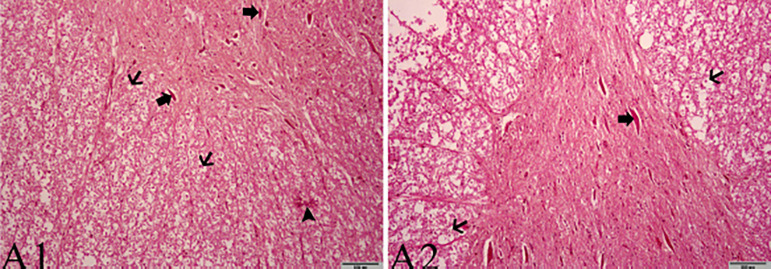
Effects of propolis on the damage caused by ischemia/reperfusion of the spinal cords of rabbits. All shapes are stained with HE. (A2) 10x and 200 µm VE (A1) 20x and 100 µm were used as the original magnification. A1: Thick arrows: chromatolysis and loss of Nissl granules in neurons. Thin arrow: formations of diffuse spherical shape. Arrowhead: focal mononuclear cell infiltration areas. A2: Thick arrow: chromatolysis and loss of Nissl granules in multipolar neurons. Thin arrows: spherulite formations.

**Note: cells:**
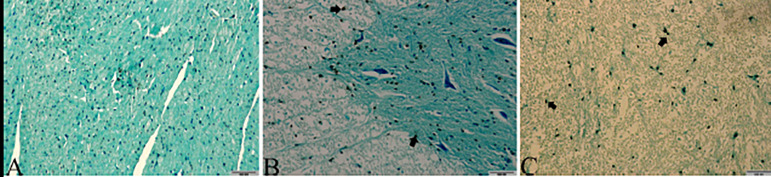
stained brown show TUNEL-positive cells (arrow). (A), (B) 10x and 200 µm were used as the original magnification.

In the histopathological examination performed by HE staining of tissue sections, there was a significant difference between the two groups in terms of chromatolysis and Nissl granule loss, formation of spherules, areas of infiltration of focal mononuclear cells (*P*=0.016, 0.027 and 0.038, respectively). There was also a difference between the two groups in terms of apoptotic index data obtained with the TUNEL method (*P*=0.001) (Table 4). In our study, the effect of propolis on the damage caused by ischemia/reperfusion in the spinal cord of rabbits can be seen histopathologically in Figures 1 and 2.

**Table 4:** Histopathological evaluation of propolis in spinal cord ischemia/reperfusion injury in rabbits.

	Histopathological finding	I/R		I/R+Propolis		F	*P*
		Mean	SD	Mean	SD		
Spinal cord	Chromatolysis and Nissl granule loss	2.43	1.03	0.90	0.80	1.553	0.016
	Spherule formations	2.43	1.03	0.88	1.03	0.001	0.027
	Focal MCI areas	1.58	0.87	0.55	0.60	0.602	0.038
TUNEL	Apoptotic index data	0.42	0.65	0.28	0.44	0.804	0.001

IR=ischemia/reperfusion; MCI=mononuclear cell infiltration; SD=standard deviation

### Results of Transmission Electron Microscopical Analysis

**Fig. 3:**
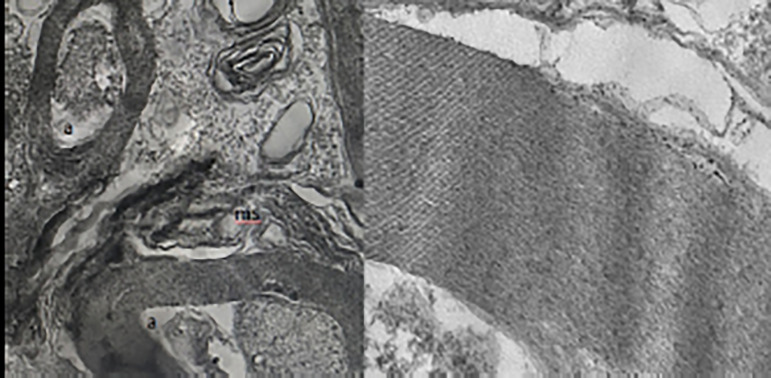
Electron microscopy examination of the spinal cord. The image on the left side shows a view of a subject from the I/R group. a: axonal/myelin separation and axoplasmic dissolution. ms: myelin separation. The image on the right side shows regular myelin sequences in the electron microscope view of a subject in the group that received propolis.

A statistically significant difference was found between the two groups (*P*=0.011) (Table 1). In the samples from the I/R group, axon-myelin separation, separation between myelin layers, complete loss of myelin integrity, withdrawal of axoplasm, mitochondria swelling and edema in cells was determined, whereas myelin, axon and the rest of cells remained sturdy in the propolis group (Figure 3).

## DISCUSSION

In the literature review, we found only two publications investigating the effect of propolis on the spinal cord ^[17,18]^. Only one of them examined the relationship between propolis and ischemia/reperfusion injury ^[17]^. On the other hand, the propolis used in these studies was not the Anzer propolis. In these studies, light microscopy and electron microscopy were not performed together.

The Anzer propolis used in this study is exclusive to the Eastern Black Sea region of Turkey; it is a type of regional propolis. The chemical composition of propolis varies depending on the vegetation, climate, season and environmental conditions of the area where it is collected.

We could not find a definitive study on the superior properties of Anzer propolis. These properties are attributed to the greater superiority of Anzer honey, which is its high ascorbic acid content compared to other honeys. However, in our literature review, we did not find a study investigating the effect of Anzer propolis on any tissue ischemia/reperfusion injury.

Inflammatory processes are among the most critical mechanisms responsible for ischemia/reperfusion damage to the spinal cord. Inflammatory cells, such as neutrophils, macrophages and monocytes, play an important role in the inflammatory response of the spinal cord to ischemic injury, causing the development of neuronal damage after reperfusion. In addition, it has been suggested that microglia, which has recently played an important role in ischemia/reperfusion injury, produces various cytotoxic mediators, such as ROS and inflammatory cytokines (IL-1, IL-6, TNF-α) ^[19]^.

In our study, at the 48^th^ hour after the procedure, the I/R group was determined to be superior to the Pr group, and there was a significant difference between the two groups in terms of IL-6 levels (*P*=0.022). Tumor necrosis factor is a chemotactic agent for alpha monocytes and polymorphonuclear leukocytes. TNF-α blockade has been shown to providing neuroprotection and improving functional recovery following experimental spinal ischemia ^[20]^. In our study, TNF-α was higher at the 48^th^ hour in the I/R group (*P*=0.040). ICAM-1 is known as the intercellular adhesion molecule-1. Its main task is to cause stable adhesion of inflammatory cells to the vascular surface. While many cells, such as endothelial cells, lymphocytes and monocytes, are normally found in small amounts, they are increased by the action of cytokines such as IL-1, TNF-α, and IFN-gamma, especially in acute or chronic inflammatory conditions. For example, melatonin reduces the synthesis of adhesion molecules P-selectin and ICAM, and shows beneficial effects by reducing cardiac damage during ischemia/reperfusion ^[21]^. In our study, the I/R group’s score was higher than the Pr group’s score at the 48^th^ hour (252,1100±15,60926b *vs*. 205,7600±14,84168b). This difference at the 48^th^ hour was statistically significant (*P*=0.012).

During ischemia/reperfusion, oxidative processes that arise from free radicals can cause protein breakdown, lipid peroxidation, and DNA damage. The endogenous antioxidant system reduces these damages through enzymatic substances such as glutathione peroxidase and superoxide dismutase. They show their effects by converting free radicals into more harmless compounds before interacting with biologically important molecules or preventing the radical production from other molecules ^[22]^. In our study, we examined TOS and TAS capacity, which we think is more effective than these markers one by one. It is possible to find different studies on the subject in the literature. In our study, TOS capacity was higher in the I/R group at the 24^th^ and 48^th^ hours after the procedure than in the Pr group, but it was only statistically significant at the 48^th^ hour (*P*=0.099 and 0.002, respectively). In contrast, TAS (mmol/ml) was higher in the Pr group 24 and 48 hours after the procedure. However, the difference between the two groups was not significant (*P*=0.833 and 0.241). In terms of TAS, there was no difference in terms of time for the groups (IR group, *P*=0.810 *vs*. Pr group, *P*=0.620) (Table 3). In addition, high circulating MPO levels are associated with inflammation and increased oxidative stress, showing the inflammatory response such as TNF-α and IL-6. MPO activity is a reliable indicator of neutrophil invasion in injured tissues. In addition, as the number of neutrophils entering the spinal cord increases, their release increases ^[23]^. The MPO enzyme is abundant in neutrophils and forms hypochlorous acid from hydrogen peroxide. In our study, the mean MPO levels were higher in the I/R group at the 24^th^ and 48^th^ hours after the procedure compared to the Pr group and there was a significant difference between the two groups (*P*=0.037 and 0.023, respectively).

Apoptosis is a programmed cell death and an event in which the cell activates several metabolic and physiological processes to self-destruct. In recent years, apoptosis has been found to be associated with some pathological conditions. Spinal cord injury involves apoptotic death of neurons after injury, which can be aggravated by inflammation. The pathological outcome can be improved by rescuing apoptotic neurons. In our study, apoptosis was less observed in the spinal cord tissue after ischemia/reperfusion in the group that was given propolis by the TUNEL method (*P*=0.001).

We used TEM in our study. There was a statistically significant difference between the groups (*P*=0.011). We found significant corruption in small, medium and large myelinated axons after ischemia/reperfusion in the untreated group. In general, there were axon-myelin separation, axoplasmic dissolution and myelin separation into different sections. However, in the treatment group that received propolis, there were axonal contractions and curlings, and a normal-looking small myelinated axon appearance and regular myelinated fiber sequences.

In our literature review, Tarlov scoring was generally used for postoperative neurological evaluation in studies related to ischemia/reperfusion in the spinal cord. For example, in a study investigating the protective efficacy of methylprednisolone and tetracosactide (ACTH1-24) in rabbit spinal cord ischemia/reperfusion injury, the mean Tarlov score of the ischemia group was significantly lower than the control group (*P*<0.001). In all cases, the mean Tarlov score in the methylprednisolone group was significantly higher than the ischemia group (*P*=0.002). The mean Tarlov score in the ACTH group was also reported to be significantly higher than that in the ischemia group (*P*<0.001). In addition, no significant difference was found in Tarlov scores between methylprednisolone and ACTH groups (*P*=0.231) ^[24]^. In our study, ischemia resulted in paraplegia at the 48^th^ hour in all animals in the reperfusion group. Propolis, which was applied before I/R damage, provided increased neurological capacity as Tarlov scores determined. After the 48^th^ hour after the procedure, a statistically significant difference was found between the two groups in terms of Tarlov score (*P*=0.012). The clinical observations we made in our study are confirmed by spinal histology.

The antioxidant capacity of propolis has been linked to caffeic acid phenethyl ester (CAPE) in some studies and to pinocebrine in others ^[25,26]^. In our study, it was not possible to say exactly which of the substances is effective and responsible for the results obtained. However, we think that ethyl oleate and 4H-1-benzopyran-4-one, which we found as a result of the analysis, could be responsible. It is possible to find studies in the literature researching the effect of these two substances. Wu et al. applied puerarin, a natural isoflavone extracted from puerarin type and contains 4H-1-benzopyran-4-one, intraperitoneally to mouse models with sciatic nerve damage. Medium and high doses of puerarin upregulated the expression of growth-associated protein 43 in L4-6 segments of the spinal cord of mice at 1^st^, 2^nd^ and 4^th^ weeks. After weeks of modeling, it reduced the atrophy of the triceps on the damaged side, and supported regeneration of the damaged spinal cord fibers after 8 weeks ^[27]^. Green et al. ^[28]^ determined, in their experimental study, that the neuroprotective effect of ethyl docosahexaenoate is due to brain tissue’s sweep ability over free radicals, and its ability to prevent the spreading of brain lipid peroxidation.

## CONCLUSION

Although it is predicted that ischemic damage can be reduced with early diagnosis, reperfusion injury and its consequences are still inevitable in this approach. For this reason, studies for the prevention of reperfusion injury have gained great importance today. Although substantial progress has been made in basic research on the mechanism of spinal cord ischemia/reperfusion injury, symptomatic therapy remains the primary treatment plan. Thereby, our studies provide evidence that, at least in one animal model, propolis can reduce IL-6, TNF-α and MPO levels, has strong antioxidant effects by measuring TAS and TOS activity, and provides inhibition of cell apoptosis in the spinal cord.

### Limitations of the Study

There are several limitations of this study. The number of animals included per group is low. There was no control group in our study. The mechanism of the protective effect of propolis on the spinal cord with a single dose is not clear in our study. In addition, different dosage regimens with more detailed grouping could be used. Also, the study did not address the possibility of cerebrospinal fluid drainage and its effects. In further studies involving more subjects, more comprehensive results can be obtained by increasing the functional, biochemical and histopathological evaluation times.

**Table:** 

Authors' roles & responsibilities	
MG	Substantial contributions to the conception or design of the work; or the acquisition, analysis, or interpretation of data for the work; drafting the work or revising it critically for important intellectual content; final approval of the version to be published
ZKS	Substantial contributions to the conception or design of the work; or the acquisition, analysis, or interpretation of data for the work; drafting the work or revising it critically for important intellectual content; final approval of the version to be published
HHD	Substantial contributions to the conception or design of the work; or the acquisition, analysis, or interpretation of data for the work; drafting the work or revising it critically for important intellectual content; final approval of the version to be published
AB	Substantial contributions to the conception or design of the work; or the acquisition, analysis, or interpretation of data for the work; drafting the work or revising it critically for important intellectual content; final approval of the version to be published
TBS	Substantial contributions to the conception or design of the work; or the acquisition, analysis, or interpretation of data for the work; drafting the work or revising it critically for important intellectual content; final approval of the version to be published
FG	Substantial contributions to the conception or design of the work; or the acquisition, analysis, or interpretation of data for the work; drafting the work or revising it critically for important intellectual content; final approval of the version to be published
NB	Substantial contributions to the conception or design of the work; or the acquisition, analysis, or interpretation of data for the work; drafting the work or revising it critically for important intellectual content; final approval of the version to be published
